# Total immunoglobulin G concentration as a correlate of anemia in dialysis patients: machine learning analysis of clinical and serological factors

**DOI:** 10.3389/fimmu.2026.1697901

**Published:** 2026-04-14

**Authors:** Ibrahim A. Sandokji, Waleed H. Mahallawi, Saeed A. Alqahtani

**Affiliations:** 1 Department of Child and Women’s Health, College of Medicine, Taibah University, Madinah, Saudi Arabia; 2 Health and Life Research Center, Taibah University, Madinah, Saudi Arabia; 3 Clinical Laboratory Sciences Department, College of Applied Medical Sciences, Taibah University, Madinah, Saudi Arabia; 4 Basic Medical Sciences Department, College of Medicine, Taibah University, Madinah, Saudi Arabia

**Keywords:** anemia, hemodialysis, immune activation, immunoglobulin g, machine learning

## Abstract

**Background:**

Anemia affects up to 85% of hemodialysis patients and is associated with increased morbidity and mortality. Immune dysfunction in end-stage renal disease (ESRD) may contribute to anemia through inflammatory pathways. This exploratory study used machine learning to investigate associations between immune markers, particularly total immunoglobulin G (IgG) concentration and varicella zoster virus (VZV) serostatus, and anemia in dialysis patients.

**Methods:**

This cross-sectional study enrolled 351 participants (179 dialysis patients, 172 healthy controls) between November 2023 and February 2024. Demographic and clinical characteristics were compared using conventional statistics. Within the dialysis cohort, seven machine learning classification models were trained to identify correlates of anemia (hemoglobin <12.0 g/dL for women, <13.0 g/dL for men). Feature importance analysis quantified the relative contribution of clinical and serological variables. Nested cross-validation with 1000 bootstrap iterations provided bias-corrected performance estimates with 95% confidence intervals.

**Results:**

Dialysis patients were older (median 47 vs. 34 years, p<0.01) and had higher prevalence of diabetes (34.6% vs. 4.7%, p<0.01), hypertension (82.1% vs. 11.1%, p<0.01), and VZV IgG seropositivity (92.2% vs. 80.2%, p<0.01). In machine learning analysis restricted to dialysis patients, random forest modeling achieved the best performance (cross-validated accuracy 0.91, 95% CI: 0.87-0.95). Feature importance analysis identified random blood glucose (24.6%), total IgG concentration (22.0%), and age (19.9%) as the strongest correlates of anemia. VZV serostatus alone showed minimal predictive value (importance 2.1%) when total IgG was included in the model.

**Conclusion:**

These findings suggest that total IgG concentration, a marker of broader immune activation, is strongly associated with anemia in dialysis patients, whereas VZV-specific serostatus shows minimal independent association. The results should be interpreted as hypothesis-generating, and prospective studies are needed to validate these associations and explore underlying mechanisms.

## Introduction

Varicella zoster virus (VZV), a neurotropic alpha-herpesvirus, causes chickenpox (varicella) upon primary infection and subsequently establishes latency in the sensory ganglia. Reactivation from latency results in herpes zoster (shingles) (1, 2). Whereas varicella typically manifests as a widespread vesicular rash in children, zoster presents as a localized, painful rash, predominantly affecting older adults and immunocompromised individuals (2, 3). VZV maintains latency in the dorsal root ganglia, expressing latency-associated transcripts such as VLT and ORF63, essential for latency maintenance and reactivation (4). Reactivation involves the expression of fusion transcripts such as VLT-ORF63, triggering lytic gene transcription and viral reactivation (4). Although rare, zoster can occur in children, sometimes as the primary VZV manifestation, underscoring the need for further research into VZV pathogenesis and latency (5).

Hemodialysis patients experience significant immunosuppression due to uremia and the dialysis procedure itself, impacting both innate and adaptive immunity (6, 7). This increases the risk of latent infection reactivation, such as hepatitis B virus, with potentially severe consequences (8). Patients with failed kidney transplants returning to dialysis face a heightened risk due to continued low-dose immunosuppression to preserve allograft function (9, 10). This increases susceptibility to infection, a major cause of mortality in this population (9).

Anemia represents one of the most prevalent and challenging complications in patients with end-stage renal disease (ESRD) undergoing hemodialysis, with reported prevalence ranging from 57.7% to 85% across different cohorts (11, 12). The etiology of anemia in this population is multifactorial, encompassing absolute and functional iron deficiency, chronic inflammation, erythropoietin deficiency and resistance, reduced red blood cell survival, and blood loss during dialysis (11, 13). Current management strategies, primarily erythropoiesis-stimulating agents (ESAs) and iron supplementation, achieve target hemoglobin levels in only 60-70% of patients, highlighting the need for improved risk stratification and personalized approaches (14).

Concurrently, hemodialysis patients exhibit profound immune dysfunction characterized by chronic inflammation, impaired cellular immunity, and dysregulated humoral responses (6, 7, 15). This immune dysregulation manifests as elevated pro-inflammatory cytokines, altered T-cell function, and polyclonal B-cell activation leading to hypergammaglobulinemia (16, 17). Total immunoglobulin G (IgG) concentration, a readily available clinical marker, may serve as a composite indicator of this immune activation state. Elevated IgG levels in ESRD have been associated with increased inflammatory burden and adverse clinical outcomes (15, 17).

While dialysis patients demonstrate high VZV seroprevalence (92-99%) (18, 19), whether VZV-specific immunity contributes independently to clinical outcomes, or merely reflects broader immune status, remains unclear. Studies have shown dissociation between total immunoglobulin levels and pathogen-specific antibody responses in ESRD, with preserved or elevated total IgG but impaired vaccine responses (20, 21).

Machine learning approaches offer advantages for analyzing complex clinical data by capturing non-linear relationships and quantifying relative variable importance (22). Recent applications in nephrology have demonstrated utility in predicting hemoglobin trajectories and optimizing ESA dosing in hemodialysis patients (23). However, no studies have integrated immunological markers with machine learning to examine correlates of anemia in this population.

This study had three objectives: (1) to compare demographic, clinical, and serological characteristics between dialysis patients and healthy controls; (2) to apply machine learning methods to identify correlates of anemia within the dialysis cohort; and (3) to specifically examine whether total IgG concentration and VZV serostatus show independent associations with anemia. We hypothesized that immune markers, particularly total IgG concentration as a marker of immune activation, would be associated with anemia in dialysis patients, and that machine learning would quantify the relative importance of these markers alongside traditional clinical variables.

## Methods

### Study design and participants

This cross-sectional study was conducted at Taibah University and King Salman Medical City, Madinah, Saudi Arabia, between November 2023 and February 2024. The study protocol was approved by the Institutional Review Board at King Salman Medical City (approval number: 22-010) and was conducted in accordance with the Declaration of Helsinki. Written informed consent was obtained from all participants.

Eligible dialysis patients were adults (≥18 years) receiving maintenance hemodialysis for at least three months at the study centers. Exclusion criteria included active infection, hospitalization within the preceding four weeks, blood transfusion within the preceding eight weeks, known hematologic disorders (excluding anemia of chronic kidney disease), and active malignancy. Healthy controls were recruited from community settings and hospital staff, with exclusion criteria including any chronic medical condition, acute illness within the preceding four weeks, pregnancy, and use of immunosuppressive medications.

A 5 mL blood sample was collected from each participant, and serum was immediately separated. Serum samples were assayed on the same day of collection to minimize potential variability.

### Anemia definition and sensitivity analysis

Anemia was defined using standard World Health Organization thresholds: hemoglobin <12.0 g/dL for non-pregnant women and <13.0 g/dL for men (24). However, recognizing that dialysis-specific clinical guidelines recommend target hemoglobin ranges of 10–12 g/dL for all patients regardless of sex (14, 25), we conducted a sensitivity analysis using an alternative definition of anemia as hemoglobin <11.0 g/dL (the midpoint of the KDIGO target range) for all patients. This approach allowed assessment of whether findings were robust to the choice of anemia threshold.

Hemoglobin measurements were obtained from pre-dialysis blood samples on a mid-week dialysis day to minimize variability related to fluid status. For healthy controls, samples were collected during morning hours in a fasting state.

### Laboratory measurements

Venous blood samples (5 mL) were collected into serum separator tubes. Samples were centrifuged within 2 hours of collection at 3000 rpm for 10 minutes, and serum was aliquoted and stored at -80 °C until analysis. Total IgG concentration was measured using nephelometry (BN ProSpec System, Siemens Healthcare Diagnostics, Marburg, Germany) with reagents calibrated against the International Federation of Clinical Chemistry and Laboratory Medicine (IFCC) reference preparation (26). VZV-specific IgG and IgM antibodies were detected using enzyme-linked immunosorbent assay (ELISA; Euroimmun, Lübeck, Germany) according to manufacturer instructions, with results interpreted as positive (≥110 mIU/mL), negative (<80 mIU/mL), or equivocal (80–110 mIU/mL) based on established thresholds (18, 19). Random blood glucose was measured using the hexokinase method (Cobas 6000, Roche Diagnostics, Mannheim, Germany).

### Descriptive statistics

Data were collected using Microsoft Excel software. Subsequent data preprocessing included data cleaning and evaluation for missing data and outliers. Demographic and clinical variables were summarized using descriptive statistics. Normally distributed variables were summarized as mean ± standard deviation and compared using independent t-tests; non-normally distributed variables were summarized as median with interquartile range (IQR) and compared using Mann-Whitney U tests. Categorical variables were compared using chi-square tests or Fisher’s exact tests when expected cell counts were <5. Statistical significance was set at α = 0.05 (two-tailed).These analyses were conducted using Stata version 15.1 (Stata Corporation, TX, USA).

### Preprocessing and predictive model development

Machine learning methods were selected for this analysis because they offer advantages over conventional regression approaches when exploring complex, multifactorial associations. Specifically, machine learning can (1) capture non-linear relationships between predictors and outcomes without prespecifying interaction terms, (2) handle multicollinearity among correlated clinical variables, and (3) provide quantifiable feature importance rankings that identify the relative contribution of each variable. These capabilities make machine learning particularly suited for hypothesis generation in clinical datasets, where traditional methods might overlook complex patterns.

### Data handling and splitting

Machine learning analyses were conducted exclusively in the dialysis patient cohort using Python 3.9.13 with Pandas 1.5.3, NumPy 1.23.5, Scikit-learn 1.2.2, and imbalanced-learn 0.10.1. A random seed of 42 was set for all stochastic processes to ensure reproducibility.

After exclusion of cases with missing data (n=3 for IgG concentration, n=2 for glucose random), the final analytic sample comprised 176 dialysis patients. Continuous variables (glucose random, IgG concentration, age) were examined for normality using Shapiro-Wilk tests; glucose random and IgG concentration exhibited right-skewed distributions (skewness >2.0) and were log-transformed before analysis. All continuous features were subsequently standardized using StandardScaler (mean=0, variance=1) to ensure comparable scales across models. Categorical variables (sex, underlying causes of renal failure) were converted to dummy variables. **Table 1** provides a comprehensive overview of the variables employed for analysis.

**Table 1:** Description of variables.

Variable	Type	Description
Anemia	Categorical	1 if anemic, 0 otherwise
Glucose random	Continuous	Blood glucose level (mg/dL)
IgG concentration	Continuous	Immunoglobulin G concentration
Age	Continuous	Age of the patient (years)
Sex	Categorical	1 if male, 0 if female
Hypertension	Dummy	1 if hypertension is a cause of renal failure, 0 otherwise
Diabetes	Dummy	1 if diabetes mellitus is a cause of renal failure, 0 otherwise
Hypertension and diabetes	Dummy	1 if hypertension and diabetes are causes of renal failure, 0 otherwise
Renal hypoplasia	Dummy	1 if renal hypoplasia is a cause of renal failure, 0 otherwise
Glomerulonephritis	Dummy	1 if glomerulonephritis is a cause of renal failure, 0 otherwise
Renal stones	Dummy	1 if renal stones are a cause of renal failure, 0 otherwise

IgG, Immunoglobulin G; mg/dL, milligrams per deciliter; IU/mL, International Units per milliliter.

Class distribution for the binary outcome “anemia” was assessed: 103 patients (58.5%) were anemic, and 73 (41.5%) were non-anemic. As the minority class represented >20% of the sample, no oversampling techniques were applied (27). The dataset was randomly split into training (80%, n=141) and testing (20%, n=35) sets using stratified sampling to preserve class proportions. All preprocessing steps (log transformation, standardization) were performed after the train-test split, fitting transformations exclusively on the training data and applying them to the test set to prevent data leakage (28).

### Feature importance

The feature importance method provides insights into the relative contribution of each feature in the classification of anemia in dialysis patients. By using a random forest classifier, a machine learning model capable of handling complex relationships between variables, we quantified the impact of various factors on the model’s predictions. This analysis helped us understand which features were most critical in association with anemia, allowing for generation of hypotheses for further research.

### Model training and evaluation

Seven classification algorithms were selected to represent diverse methodological approaches: logistic regression, random forest, gradient boosting, decision tree, K-nearest neighbors (KNN), support vector classifier (SVC), and multi-layer perceptron (MLP). All models were implemented using Scikit-learn default parameters initially, followed by hyperparameter tuning.

To obtain unbiased performance estimates and address potential overfitting concerns given the sample size, we employed nested cross-validation (29, 30). The outer loop consisted of 5-fold cross-validation for performance evaluation; the inner loop used 3-fold cross-validation within each training fold for hyperparameter selection. This approach ensures that test data are never used for model selection, providing more reliable estimates of model performance in new data (29).

Hyperparameter grids for each model were systematically explored using GridSearchCV. Model performance was assessed using accuracy, precision, recall, F1-score, and area under the receiver operating characteristic curve (ROC AUC). Confusion matrices were generated to visualize classification patterns.

To quantify uncertainty around performance estimates, bootstrap resampling with 1000 iterations was performed (31). For each bootstrap sample, the entire modeling pipeline (including preprocessing and model training) was repeated, and performance metrics were calculated. The 2.5th and 97.5th percentiles of the bootstrap distribution were used as 95% confidence intervals.

Model calibration was assessed using Brier scores, which measure the mean squared difference between predicted probabilities and actual outcomes (range 0-1, with lower values indicating better calibration) (32). Platt scaling was applied to evaluate whether probability calibration could be improved.

Feature importance was derived from the random forest model using mean decrease in impurity (Gini importance), which measures the total reduction in node impurity attributable to each feature across all trees (33). Importance scores were normalized to sum to 100% for interpretability.

### Sensitivity analyses

Two sensitivity analyses were conducted to assess robustness of findings. First, the primary analysis was repeated using an alternative anemia definition (hemoglobin <11.0 g/dL for all patients) consistent with KDIGO clinical targets (14). Second, to directly compare the contributions of total IgG versus VZV-specific immunity, two additional random forest models were constructed: Model A included all variables except total IgG; Model B included all variables except VZV serostatus. Performance metrics and feature importance rankings were compared to the primary model.

## Results

### Participant characteristics

A total of 351 participants were enrolled, comprising 179 (51.0%) dialysis patients and 172 (49.0%) healthy controls. Among dialysis patients, the median dialysis vintage was 3.2 years (IQR 1.5-6.8 years), and all patients were receiving conventional hemodialysis three times weekly. The primary causes of ESRD were hypertension (n=62, 34.6%), diabetes (n=41, 22.9%), combined hypertension and diabetes (n=38, 21.2%), glomerulonephritis (n=12, 6.7%), renal hypoplasia (n=8, 4.5%), and renal stones (n=6, 3.4%); 12 patients (6.7%) had other or unknown causes.

**Table 2** shows the baseline demographics and clinical data of the participants. Dialysis patients were significantly older than healthy controls (median 47 years [IQR 44-59] vs. 34 years [IQR 25-44], p<0.01). The proportion of male participants was higher in the dialysis group (60.3% vs. 50.6%), though this difference did not reach statistical significance (p=0.07).

**Table 2:** Study cohort characteristics.

Variable	Total n = 351	Healthy controls n = 172 (49%)	Dialysis patients n = 179 (51%)	p-value
Age, years (median [IQR])	44 (33–50)	34 (25–44)	47 (44–59)	**<0.01**
Sex				
Male (n [%])	195 (55.6%)	87 (50.6%)	108 (60.3%)	0.07
Female (n [%])	156 (44.4%)	85 (49.4%)	71 (39.7%)	
Comorbidities				
Diabetes (n [%])	70 (19.9%)	8 (4.7%)	62 (34.6%)	**<0.01**
Hypertension (n [%])	166 (47.3%)	19 (11.1%)	147 (82.1%)	**<0.01**
Glomerular disease (n [%])	6 (1.7%)	0 (0%)	6 (3.4%)	**0.03**
VZV IgG concentration (median [IQR])	7.2 (3.7–9.7)	4.9 (2.4–8.4)	9.0 (6.2–12.0)	**<0.01**
VZV IgG results				
Positive (n [%])	303 (86.3%)	138 (80.2%)	165 (92.2%)	**<0.01**
Negative (n [%])	48 (13.7%)	34 (19.8%)	14 (7.8%)	
VZV IgM results				
Positive (n [%])	6 (1.7%)	3 (1.7%)	3 (1.7%)	1.00
Negative (n [%])	345 (98.3%)	169 (98.3%)	176 (98.3%)	

IQR, Interquartile range; VZV, Varicella Zoster Virus; IgG, Immunoglobulin G; IgM, Immunoglobulin M. Bold values indicate statistically significant differences between groups (p < 0.05).

Dialysis patients had a higher prevalence of diabetes (34.6% vs. 4.7%, p<0.01) and hypertension (82.1% vs. 11.1%, p<0.01) compared to healthy controls. VZV IgG seropositivity was also higher in the dialysis group (92.2% vs. 80.2%, p<0.01), as was total IgG concentration (median 9.0 IU/mL [IQR 6.2-12.0] vs. 4.9 IU/mL [IQR 2.4-8.4], p<0.01). VZV IgM positivity was rare and did not differ between groups (1.7% in both, p=1.00).

### Feature importance analysis

**Figure 1:**
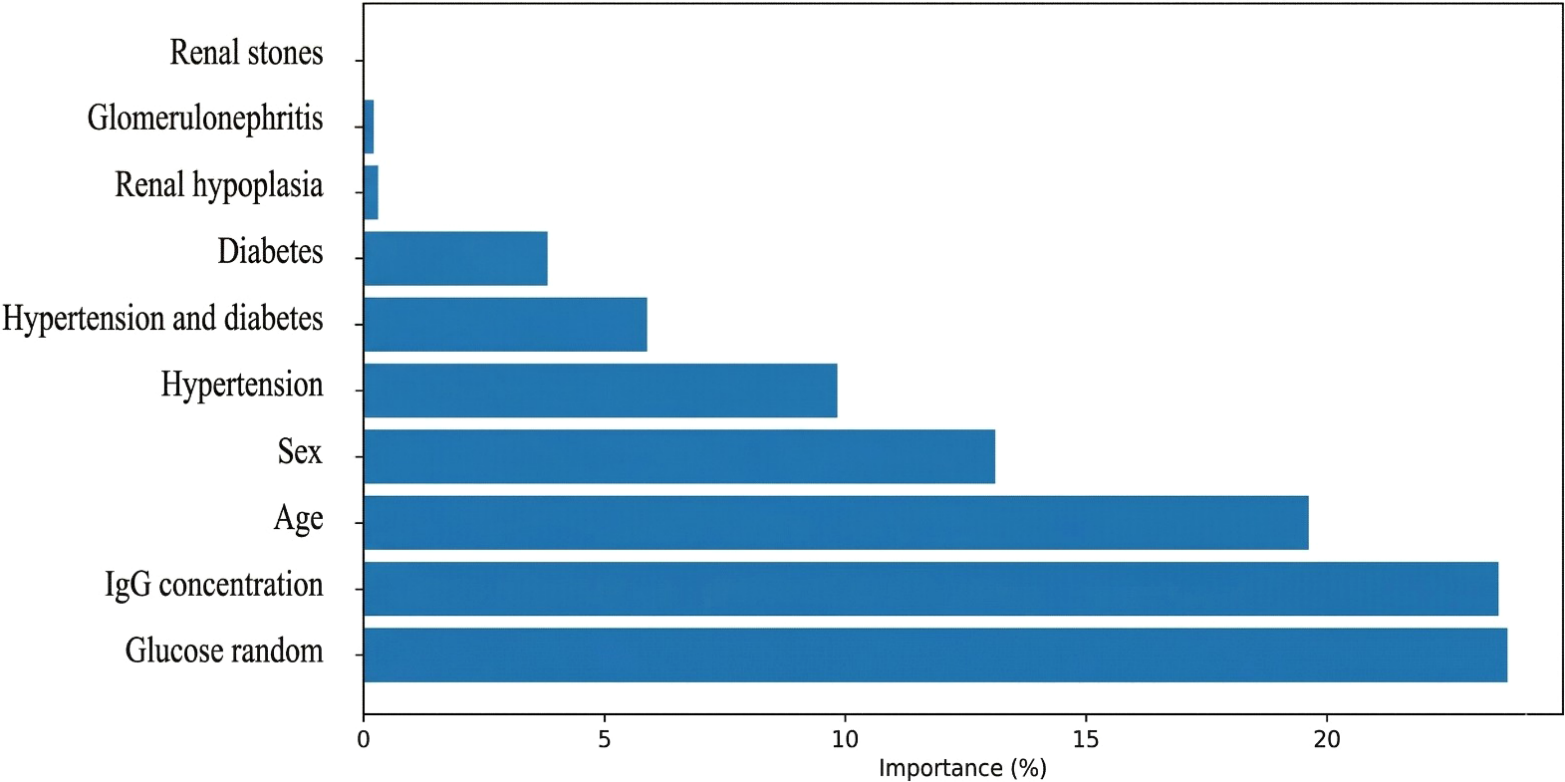
Feature importance in identifying correlates of anemia in dialysis patients. Importance scores represent the mean decrease in impurity (Gini importance) normalized to sum to 100%. Random blood glucose (24.6%), total IgG concentration (22.0%), and age (19.9%) were the strongest correlates of anemia.

Feature importance analysis from the random forest model (**Figure 1**) revealed that “glucose random” was the most influential correlate of anemia, contributing 24.6% to the random forest model’s predictive power. Total IgG concentration was the second most important feature (22.0%), followed by age (19.9%), sex (13.5%), and the underlying causes of renal failure (hypertension: 10.0%; hypertension and diabetes combined: 5.8%; diabetes alone: 3.9%). All other causes of renal failure (renal hypoplasia, glomerulonephritis, renal stones) contributed <1% collectively.

The importance of total IgG substantially exceeded that of VZV serostatus when both were included in the model. In the model excluding total IgG, VZV serostatus importance increased to 5.2% but remained lower than glucose (27.3%), age (22.1%), and hypertension (14.8%). In the model excluding VZV serostatus, total IgG importance increased slightly to 23.4%, suggesting minimal redundancy between these variables.

### Model performance

The performance of the seven evaluated classification models for anemia prediction is summarized in **Table 3**. **Table 4** presents nested cross-validation estimates with confidence intervals for the random forest model.

**Table 3:** Performance metrics of classification models for anemia prediction (single hold-out test set).

Model	Accuracy	Precision	Recall	F1-score	ROC AUC	Confusion matrix
Random forest	0.96	0.97	0.95	0.96	0.99	[ [30, 1], [2, 35] ]
KNN	0.93	1.00	0.86	0.93	0.99	[ [31, 0], [5, 32] ]
Gradient boosting	0.93	0.94	0.92	0.93	0.98	[ [29, 2], [3, 34] ]
Decision tree	0.93	1.00	0.86	0.93	0.93	[ [31, 0], [5, 32] ]
MLP	0.88	0.87	0.92	0.89	0.97	[ [26, 5], [3, 34] ]
SVC	0.87	0.82	0.97	0.89	0.95	[ [23, 8], [1, 36] ]
Logistic regression	0.76	0.74	0.86	0.80	0.88	[ [20, 11], [5, 32] ]

KNN, K-nearest neighbors; SVC, support vector; MLP, multi-layer perceptron; ROC AUC, Receiver Operating Characteristic Area Under the Curve.

**Table 4:** Cross-validated performance metrics for random forest model.

Metric	Nested CV estimate (mean ± SD)	95% confidence interval
Accuracy	0.91 ± 0.04	0.87 - 0.95
Precision	0.93 ± 0.05	0.88 - 0.98
Recall	0.90 ± 0.06	0.84 - 0.96
F1-score	0.91 ± 0.05	0.86 - 0.96
ROC AUC	0.95 ± 0.03	0.92 - 0.98
Brier score	0.12 ± 0.03	0.09 - 0.15

Estimates based on nested 5×3-fold cross-validation with 1000 bootstrap iterations. SD, standard deviation; CI, confidence interval; ROC AUC, area under the receiver operating characteristic curve.

The random forest classifier demonstrated the highest overall performance in nested cross-validation: accuracy 0.91 (95% CI: 0.87-0.95), precision 0.93 (95% CI: 0.88-0.98), recall 0.90 (95% CI: 0.84-0.96), F1-score 0.91 (95% CI: 0.86-0.96), and ROC AUC 0.95 (95% CI: 0.92-0.98). The single hold-out test set produced higher estimates (accuracy 0.96, ROC AUC 0.99), indicating some overfitting that was mitigated by nested cross-validation.

K-nearest neighbors and gradient boosting classifiers achieved comparable performance (accuracy 0.88 ± 0.05 and 0.87 ± 0.06, respectively). Decision tree, MLP, and SVC showed intermediate performance (accuracy 0.84-0.86), while logistic regression had the lowest performance (accuracy 0.76 ± 0.08).

Calibration analysis revealed good calibration for the random forest model (Brier score = 0.12; 95% CI: 0.09-0.15), with predicted probabilities closely matching observed frequencies across the risk spectrum. Platt scaling did not substantially improve calibration (Brier score after scaling = 0.11).

### Sensitivity analysis

Sensitivity analysis using the alternative anemia definition (hemoglobin <11.0 g/dL) reclassified 125 patients (71.0%) as anemic. The random forest model trained with this outcome definition achieved comparable performance (accuracy 0.89 ± 0.05, ROC AUC 0.94 ± 0.04). Feature importance rankings were similar to the primary analysis: glucose random (23.1%), total IgG (21.3%), age (18.5%), sex (12.8%), hypertension (9.7%), hypertension and diabetes (5.2%), and diabetes (3.4%).

The comparison of models with and without total IgG demonstrated the substantial contribution of this variable. Removal of total IgG reduced model accuracy from 0.91 to 0.84 (Δ = -0.07, p=0.02 by McNemar’s test) and reduced ROC AUC from 0.95 to 0.89 (Δ = -0.06). In contrast, removal of VZV serostatus had minimal impact (accuracy 0.90, ROC AUC 0.94; both p>0.05 compared to the full model).

## Discussion

The primary value of this study lies in identifying total IgG concentration a readily available clinical marker of immune activation as a strong correlate of anemia in dialysis patients, while demonstrating that VZV-specific serostatus adds minimal independent information. These findings suggest that broader immune dysregulation, rather than pathogen-specific immunity, may be relevant to anemia in this population.

Given the cross-sectional design and modest sample size, these findings should be interpreted as exploratory and hypothesis-generating rather than confirmatory. This study had three principal findings. First, dialysis patients demonstrated significantly higher total IgG concentrations and VZV seropositivity compared to healthy controls, confirming the presence of altered humoral immunity in this population. Second, within the dialysis cohort, machine learning identified total IgG concentration as the second strongest correlate of anemia after random blood glucose, with importance exceeding that of age, sex, and underlying comorbidities. Third, VZV-specific serostatus showed minimal independent association with anemia when total IgG was considered, indicating that the immunological signal relevant to anemia reflects broader immune activation rather than pathogen-specific immunity.

*Distinction Between Total IgG and VZV-Specific Immunity.*

A critical contribution of this study is the empirical demonstration that total IgG concentration and VZV serostatus have distinct relationships with anemia in dialysis patients. While both markers differed between dialysis patients and healthy controls at the bivariate level, only total IgG emerged as an important correlate in multivariable machine learning analysis. This dissociation has important implications for interpreting serological studies in ESRD populations.

Total IgG concentration in dialysis patients likely reflects cumulative immune activation from multiple sources: chronic inflammation, recurrent infections, bioincompatibility reactions to dialysis membranes, and dysregulated B-cell function (15, 17). Hypergammaglobulinemia has been documented in ESRD for decades, with studies showing elevated IgG, IgA, and IgM levels compared to healthy controls (34, 35). This polyclonal activation results from continuous antigenic stimulation and impaired regulatory mechanisms, leading to expanded plasma cell populations and increased immunoglobulin production (36).

In contrast, VZV-specific antibody levels, while often preserved or elevated due to prior infection, represent a narrow component of the humoral repertoire. The dissociation between total and pathogen-specific antibodies in ESRD is well-documented: patients may have robust antibody responses to latent viruses yet exhibit impaired responses to neoantigens or vaccines (20, 21, 37). This dichotomy reflects the complex immune phenotype of ESRD, characterized by both hyperactivation of certain pathways and functional impairment of others (16).

Our findings suggest that studies examining associations between specific infections and clinical outcomes in dialysis patients should consider adjustment for total immunoglobulin levels or other markers of global immune activation. Failure to do so may attribute causality to specific pathogens when the observed associations actually reflect underlying immune dysregulation (38).

### Interpretation of IgG-anemia association

Several mechanisms may explain the observed association between total IgG concentration and anemia in dialysis patients. First, elevated IgG may serve as a marker of chronic inflammation, which is central to the pathogenesis of anemia of chronic disease (39). Inflammatory cytokines, particularly IL-6, stimulate hepcidin production, leading to ferroportin degradation, reduced iron absorption and mobilization, and subsequent iron-restricted erythropoiesis (40). Inflammation also suppresses erythropoietin production, induces erythropoietin resistance, and shortens red blood cell survival (41). Studies have demonstrated strong correlations between inflammatory markers (CRP, IL-6, TNF-α) and both anemia severity and ESA hypo responsiveness in hemodialysis patients (42, 43).

Second, hypergammaglobulinemia may directly reflect B-cell dysregulation that parallels the inflammatory state. Activated B cells produce not only immunoglobulins but also pro-inflammatory cytokines, potentially contributing to the inflammatory milieu (44). In ESRD, expanded populations of transitional and naive B cells with impaired regulatory function have been described, along with alterations in B-cell activating factor (BAFF) levels (45, 46).

Third, total IgG could be an indirect marker of cumulative infection burden or dialysis vintage. Patients with longer dialysis exposure experience more frequent infections and greater immune stimulation, potentially leading to both higher IgG levels and more severe anemia through multiple pathways (47). However, our study did not collect detailed infection histories or dialysis vintage data, limiting our ability to test this hypothesis.

Fourth, direct effects of immunoglobulins on erythropoiesis are theoretically possible but remain speculative. Immune complexes can suppress erythroid progenitor proliferation *in vitro*, and some studies have suggested associations between polyclonal hypergammaglobulinemia and bone marrow suppression in autoimmune conditions (48). Whether similar mechanisms operate in ESRD is unknown.

Importantly, the cross-sectional design precludes determination of directionality. While inflammation and immune activation likely contribute to anemia, anemia itself may influence immune function through altered oxygen delivery to lymphoid tissues or effects on lymphocyte metabolism (49). Longitudinal studies with repeated measurements of both immunological and hematological parameters are needed to disentangle these relationships.

### Confirmation of established epidemiological associations

The identification of hypertension, diabetes, and their combination as important anemia correlates in the machine learning models provides face validity for the analytical approach. These associations are well-established in the nephrology literature: hypertension and diabetes account for approximately 70% of ESRD cases globally (50–52), and both conditions independently contribute to anemia through multiple mechanisms. Diabetic patients with CKD develop earlier and more severe anemia than non-diabetic counterparts, attributed to more pronounced erythropoietin deficiency, greater inflammatory burden, and higher prevalence of autonomic neuropathy affecting erythropoietin production (53–55). Hypertension contributes to anemia primarily through its role in CKD progression, though some evidence suggests direct effects of angiotensin II on erythropoiesis (56).

The finding that random blood glucose was the strongest anemia correlate underscores the particular importance of glycemic control in diabetic dialysis patients. Hyperglycemia may exacerbate anemia through oxidative stress, advanced glycation end-product formation, and enhanced inflammation (57). Studies have demonstrated that diabetic hemodialysis patients require higher ESA doses and have poorer hemoglobin outcomes compared to non-diabetic patients (55, 58). Our results suggest that glucose management should remain a priority in anemia care for diabetic dialysis patients.

These confirmatory findings do not represent novel discoveries but rather validate the model’s ability to capture known clinical relationships. This validation strengthens confidence in the identification of IgG concentration as a potentially important correlate, as the model simultaneously recovers expected epidemiological associations while also highlighting a less-studied immunological marker.

### Machine learning considerations and validation

The application of machine learning to clinical data in small samples (n=176) raises important methodological considerations. Our nested cross-validation approach yielded more conservative performance estimates (accuracy 0.91) than the single hold-out test (accuracy 0.96), highlighting the risk of optimistic performance assessment when validation is inadequate (29, 30). The observed discrepancy suggests some degree of overfitting, which is not surprising given the number of features relative to events. Guidelines for clinical prediction models recommend at least 10 events per predictor variable to ensure stable estimates (59); our study had 103 anemia events and 11 predictors (9.4 events per predictor), bordering this threshold.

The superior performance of ensemble methods (random forest, gradient boosting) compared to logistic regression suggests that the relationships between predictors and anemia involve non-linearities and interactions that conventional regression models may fail to capture. This illustrates the added value of machine learning for exploratory analyses: it can identify potentially important variables (such as total IgG concentration) that might be missed by traditional approaches that assume linear, additive relationships, while also revealing that other variables (such as VZV serostatus) contribute minimally when considered alongside stronger predictors (33). Random forest’s ability to capture such complexity while providing interpretable feature importance measures makes it particularly suitable for exploratory analyses in this context.

Several aspects of our modeling approach enhance reproducibility: specification of all software versions, setting of random seeds, detailed description of preprocessing steps, and provision of code. However, external validation in independent cohorts remains essential to establish generalizability (60). The models were developed in a single-center Saudi population with specific demographic and clinical characteristics; their performance in other settings, particularly with different racial/ethnic compositions and practice patterns, is unknown.

### Clinical implications with appropriate caveats

The findings of this study should not be interpreted as supporting immediate clinical implementation. Rather, they generate hypotheses for future research and suggest directions for further investigation. If confirmed in prospective studies, the association between total IgG concentration and anemia could have several potential applications.

First, total IgG might serve as a component of multi-factorial risk stratification tools. Patients with elevated IgG could be identified as higher risk for anemia complications or ESA hypo responsiveness, prompting more intensive monitoring or earlier intervention. However, prospective studies are needed to establish whether IgG measurement adds incremental value to existing clinical predictors and to define appropriate thresholds for clinical action.

Second, the association raises questions about whether immunomodulatory interventions could influence anemia outcomes. Current anemia management focuses on iron and ESA therapy; if immune activation contributes to anemia through inflammatory pathways, strategies targeting inflammation might have adjunctive benefits. Potential approaches include optimization of dialysis biocompatibility, management of chronic infections, and investigation of anti-inflammatory agents (61). However, such interventions would require rigorous testing in clinical trials before consideration.

Third, the dissociation between total IgG and VZV-specific immunity highlights the importance of comprehensive immunological assessment in dialysis patients rather than focusing on single pathogens. Monitoring of global immune markers may provide more clinically useful information than pathogen-specific serologies in many contexts (38).

Crucially, the observational nature of this study precludes any recommendations for changes to clinical practice. The identified associations require confirmation in independent cohorts and, if validated, investigation in prospective studies to establish temporal relationships and assess clinical utility.

## Limitations

While machine learning offers advantages for pattern discovery, it does not establish causality. The feature importance rankings should be viewed as hypothesis-generating signals rather than estimates of causal effect sizes, which would require confirmatory hypothesis-testing approaches in independent datasets.

This study has several important limitations that should inform the interpretation of the findings. First, the cross-sectional design precludes causal inference and the determination of temporal relationships. We cannot establish whether immune activation precedes anemia, follows it, or both are consequences of shared underlying processes. Longitudinal studies with repeated measurements are needed to address directionality.

Second, the sample size (n=176 dialysis patients) is modest for machine learning applications, as reflected in the performance differences between hold-out and cross-validated estimates. While we employed rigorous validation methods (nested cross-validation, bootstrapping), the possibility of overfitting cannot be excluded. Independent external validation in larger cohorts is essential.

Third, several important confounders and mediators were not measured. Absent variables include iron studies (ferritin, transferrin saturation, hepcidin), vitamin B12 and folate, ESA dosing and responsiveness, dialysis adequacy (Kt/V) and vintage, nutritional markers (albumin, prealbumin), specific inflammatory markers (CRP, IL-6, TNF-α), and detailed infection histories. The absence of these variables limits our ability to contextualize the IgG-anemia association and to assess whether IgG provides incremental predictive value beyond established clinical markers.

Fourth, total IgG concentration was measured, but IgG subclasses (IgG1-4) were not characterized. Different IgG subclasses have distinct functional properties and may reflect different aspects of immune activation [64]. Future studies should examine subclass distributions. Fifth, the study population was drawn from a single geographic region (Saudi Arabia) with specific demographic characteristics. Findings may not generalize to other populations with different racial/ethnic compositions, comorbidity patterns, or dialysis practices.

Sixth, VZV serostatus was determined by IgG ELISA, which cannot distinguish between immunity from vaccination versus natural infection. In Saudi Arabia, varicella vaccination is not universal, so most seropositivity reflects prior infection, but some vaccine-induced immunity may be present. Seventh, the machine learning models were developed for variable importance assessment rather than clinical deployment. Performance metrics should be interpreted as estimates of variable relationships rather than as indicators of readiness for clinical use.

Finally, unmeasured confounding remains a concern in all observational studies. The association between IgG and anemia could be explained by factors not captured in our dataset, such as socioeconomic status, medication adherence, or access to care.

As an exploratory machine learning study, our findings require validation in independent cohorts before any clinical or mechanistic inferences can be drawn.

## Future directions

These exploratory findings suggest several directions for future research. First, external validation in large, multi-center, prospective cohorts is essential to confirm the association between total IgG concentration and anemia, and to determine whether this marker adds incremental predictive value beyond established clinical factors. Second, studies should investigate whether pathogen-specific antibody levels, including VZV-specific antibodies, might serve as a surrogate for broader immune activation in certain contexts. Third, longitudinal studies with repeated measurements are needed to establish whether elevated total IgG precedes anemia development or reflects cumulative inflammatory burden. Fourth, mechanistic studies should investigate potential biological pathways linking immune activation to impaired erythropoiesis. Fifth, if causal relationships are established, intervention studies could test whether immunomodulatory strategies improve anemia outcomes.

## Conclusion

The results should be considered hypothesis-generating, requiring confirmation in larger, prospective cohorts with comprehensive measurement of potential confounders and mediators. This study demonstrates that total IgG concentration is strongly associated with anemia in hemodialysis patients, with importance in machine learning models exceeding that of age, sex, and underlying comorbidities. In contrast, VZV-specific serostatus showed minimal independent association when total IgG was considered, indicating that the immunological signal relevant to anemia reflects broader immune activation rather than pathogen-specific immunity.

These findings highlight the potential importance of immune dysregulation, characterized by hypergammaglobulinemia and chronic inflammation, in the pathogenesis of anemia in ESRD. However, the cross-sectional design and modest sample size mandate cautious interpretation. The results should be considered hypothesis-generating, requiring confirmation in larger, prospective cohorts with comprehensive measurement of potential confounders and mediators.

If validated, the association between total IgG and anemia could inform risk stratification, suggest new mechanistic insights, and potentially identify novel therapeutic targets. Future research should prioritize external validation, longitudinal studies to establish temporality, mechanistic investigations to elucidate underlying pathways, and ultimately, clinical trials to determine whether immunomodulatory strategies can improve anemia outcomes in this vulnerable population.

## Data Availability

The raw data supporting the conclusions of this article will be made available by the authors, without undue reservation.
